# Dissecting Genome-Wide Association Signals for Loss-of-Function Phenotypes in Sorghum Flavonoid Pigmentation Traits

**DOI:** 10.1534/g3.113.008417

**Published:** 2013-11-01

**Authors:** Geoffrey P. Morris, Davina H. Rhodes, Zachary Brenton, Punna Ramu, Vinayan Madhumal Thayil, Santosh Deshpande, C. Thomas Hash, Charlotte Acharya, Sharon E. Mitchell, Edward S. Buckler, Jianming Yu, Stephen Kresovich

**Affiliations:** * Department of Biological Sciences, University of South Carolina, Columbia, South Carolina 29208; † ICRISAT, Patancheru PO, Hyderabad 502 324, Andhra Pradesh, India; ‡ ICRISAT-Sadoré, BP 12404, Niamey, Niger; § Institute for Genomic Diversity, Cornell University, Ithaca, New York 14853; ** USDA-ARS, Department of Plant Breeding and Genetics, Cornell University, Ithaca, New York 14853; †† Department of Agronomy, Iowa State University, Ames, Iowa 50011

**Keywords:** quantitative trait loci, null alleles, structured populations, genome scan, grain pigmentation

## Abstract

Genome-wide association studies are a powerful method to dissect the genetic basis of traits, although in practice the effects of complex genetic architecture and population structure remain poorly understood. To compare mapping strategies we dissected the genetic control of flavonoid pigmentation traits in the cereal grass sorghum by using high-resolution genotyping-by-sequencing single-nucleotide polymorphism markers. Studying the grain tannin trait, we find that general linear models (GLMs) are not able to precisely map *tan1-a*, a known loss-of-function allele of the *Tannin1* gene, with either a small panel (*n* = 142) or large association panel (*n* = 336), and that indirect associations limit the mapping of the *Tannin1* locus to Mb-resolution. A GLM that accounts for population structure (Q) or standard mixed linear model that accounts for kinship (K) can identify *tan1-a*, whereas a compressed mixed linear model performs worse than the naive GLM. Interestingly, a simple loss-of-function genome scan, for genotype-phenotype covariation only in the putative loss-of-function allele, is able to precisely identify the *Tannin1* gene without considering relatedness. We also find that the *tan1-a* allele can be mapped with gene resolution in a biparental recombinant inbred line family (*n* = 263) using genotyping-by-sequencing markers but lower precision in the mapping of vegetative pigmentation traits suggest that consistent gene-level resolution will likely require larger families or multiple recombinant inbred lines. These findings highlight that complex association signals can emerge from even the simplest traits given epistasis and structured alleles, but that gene-resolution mapping of these traits is possible with high marker density and appropriate models.

Genome-wide association studies (GWAS) have been used to dissect the genetic basis of complex traits in humans (Rosenberg *et al.* 2010; Visscher *et al.* 2012), model systems (Atwell *et al.* 2010; Flint and Eskin 2012), and crop species (Beló *et al.* 2008; Cockram *et al.* 2010; Huang *et al.* 2010, 2012; Morris *et al.* 2013). By the use of natural populations instead of biparental families traditionally used in linkage mapping, GWAS offers a number of potential advantages: avoiding the need for crosses; capturing greater genetic diversity for the trait of interest; and providing greater mapping resolution through the use of historical recombination events. However, complex genetic architectures and structured traits can create spurious signals and indirect associations in GWAS, and the interactions between genetic architecture and population structure remain poorly understood. Complex genetic architecture can arise from allelic heterogeneity (multiple independent alleles at the same gene) or genetic heterogeneity (multiple genes controlling the trait), and epistasis (nonadditive interactions among multiple genes; Platt *et al.* 2010; Segura *et al.* 2012). A simple, common form of epistasis is complementary dominance, in which functional alleles of two or more genes are required for the expression of the dominant phenotype, so loss-of-function alleles interact epistatically. When multiple functional alleles are present on the same haplotype block, synthetic associations with shared ancestral alleles may result in positively misleading GWAS signals (Dickson *et al.* 2010; Platt *et al.* 2010). Complex genetic interactions are thought to underlie cases in which known causative alleles do not emerge as the most significant associations in GWAS, such as the case of *FRIGIDA* expression in Arabidopsis (Atwell *et al.* 2010; Segura *et al.* 2012).

Given the complexity of mapping traits in diverse populations, much effort has gone to developing and characterizing various statistical approaches to GWAS. It is well-known that the simplest GWAS methods that ignore population structure, such as Wilcoxon rank sum tests or general linear models (GLMs), will yield inflated association signals when used for structured traits (Atwell *et al.* 2010; Huang *et al.* 2010; Zhao *et al.* 2011). Conversely, approaches that control for inflated signals by accounting for population structure [including the structured association (Pritchard *et al.* 2000) and mixed linear models (Yu *et al.* 2006)] can yield false-negative results when causal variants are structured (Bergelson and Roux 2010). Given the trade-offs in the use of simple *vs.* structured models, further empirical studies of validated functional variants are needed to inform investigations of novel trait loci. Moreover, it has been argued that the impact and prevalence of synthetic associations will only be determined empirically (Goldstein 2011).

To better characterize the interaction of genetic architecture and population structure, and compare methods for GWAS in structured populations, we investigated the genome-wide association signals of flavonoid pigmentation traits. Flavonoid pigmentation shows abundant natural variation in many plant species and therefore has been a classic empirical model in genetics (Darwin 1859; Nilsson-Ehle 1909; Sax 1923; McClintock 1950; Huang *et al.* 2010). As such, the flavonoid pathway has been almost completely elucidated in Arabidopsis and maize, and many of the enzymes, regulators, and transporters underlying flavonoid traits are widely conserved across plant families (Petroni and Tonelli 2011; Supporting Information, Table S1). Although the core components of the flavonoid network are relatively well-understood, there are a number of areas that remain to be elucidated, including the polymerization and transport of tannins (Zhao *et al.* 2010) and pathways responsible for lineage-specific end products involved in defense and environmental adaptation (Ibraheem *et al.* 2010). Moreover, even when the genes underlying a trait are known, the characterization of functional allelic variation remains an important and challenging goal (Tian *et al.* 2009).

In sorghum [*Sorghum bicolor* (L.) Moench] there is abundant natural variation for flavonoid pigmentation, which underlies a number of agronomic traits, such as grain mold (Esele *et al.* 1993) and anthracnose resistance (Ibraheem *et al.* 2010), and nutritional traits, such as digestibility (Kaufman *et al.* 2012) and anti-inflammatory properties (Moraes *et al.* 2012). The role of pigmentation in crop diversification and improvement is complex (Gross and Olsen 2010), as exemplified by grain tannins, which provide defense against molding and bird predation but also impart bitterness and astringency (Doggett 1988). Classical inheritance and linkage studies have mapped loci controlling pigmentation of several sorghum tissues including the testa (inner seed coat; *B1* and *B2*), pericarp (outer seed coat; *R* and *Y*), coleoptile (seedling leaf sheath; *Rs1* and *Rs2*), and adult vegetative leaf and stem (*P* and *Q*) (Vinall and Cron 1921; Stephens 1946; Doggett 1988; Mace and Jordan 2010). In addition, two flavonoid genes have been cloned in sorghum, which can be used to validate mapping approaches: a MYB transcription factor (*Y1*; *Yellow seed1*), which controls pericarp pigmentation and phytoalexin production (Ibraheem *et al.* 2010), and a WD40 regulator (*Tannin1*), which controls the presence of tannins in the testa (Wu *et al.* 2012). Although testa pigmentation segregates as a simple Mendelian trait, from the standpoint of GWAS it may present a complex genetic architecture because of multiple *tan1* loss-of-function alleles (*tan1-a* and *tan1-b*) and complementary dominance between at least two loss-of-function loci (Wu *et al.* 2012). Moreover, sorghum has complex population structure because of extensive ancient crop diffusion and a propensity for inbreeding, which presents a challenge for GWAS of agroclimatic traits (Morris *et al.* 2013). Here, we take advantage of high-resolution genotyping-by-sequencing single-nucleotide polymorphism (SNP) maps to compare the ability of several genome-wide mapping approaches to identify known flavonoid pigmentation loci, contrast linear model GWAS to a simple loss-of-function genome scan, and use GWAS to identify additional loci that may underlie flavonoid pigmentation in sorghum.

## Materials and Methods

### Flavonoid-related candidate genes

To identify potential components of the flavonoid network in sorghum and to define an *a priori* candidate gene set for comparison with mapping results, we conducted a systematic survey of flavonoid-related gene families in the reference sorghum genome (Table S1 and File S1; Paterson *et al.* 2009). Because Arabidopsis is by far the best-understood model for the flavonoid network (Winkel-Shirley 2001; Zhao *et al.* 2010; Petroni and Tonelli 2011), we defined the candidate gene set primarily based on the Arabidopsis flavonoid-related genes in TAIR (www.arabidopsis.org; Lamesch *et al.* 2011). Sorghum homologs of the reference genes were obtained from Phytozome (www.phytozome.org; Goodstein *et al.* 2011). Note that because most flavonoid-related genes are conserved across diverse plant species, an Arabidopsis-based homology search captures the sorghum orthologs of many flavonoid-related genes in maize, rice, and other plant species (Schnable and Freeling 2011; Petroni and Tonelli 2011), as well as the two cloned flavonoid genes in sorghum, *Yellow seed1* and *Tannin1* (Ibraheem *et al.* 2010; Wu *et al.* 2012).

### Genotyping-by-sequencing (GBS)

Genotypes for this study were generated with genotyping-by-sequencing (Elshire *et al.* 2011) using the GBS pipeline 3.0 in the TASSEL software package (Bradbury *et al.* 2007) and the BTx623 genome as a reference (Paterson *et al.* 2009). Genotypes for the association panels at 265,487 SNPs were previously obtained (Morris *et al.* 2013). For this study we also genotyped the same 265,487 SNPs in 263 F_6-7_ recombinant inbred lines (RILs; ICSV 745 × PB 15520) that were developed as a stem borer resistance mapping population but also segregate flavonoid pigmentation phenotypes (Vinayan 2010). Note, SNP positions in the GBS data may differ slightly (several base pairs) from the reference genome because of small indel polymorphisms. Missing genotype calls were imputed using the FastImputationBitFixedWindow plugin in TASSEL 4.0 (http://sourceforge.net/projects/tassel/).

### Pigmentation phenotypes

Tannin phenotypes for the small association panel (*n* = 142) were previously published (Wu *et al.* 2012). This panel represents a subset of early-maturing, semi-dwarf accessions from the U.S. Sorghum Association Panel (Casa *et al.* 2008). For the large association panel, seeds for the full Sorghum Association Panel were obtained from the U.S. National Plant Germplasm System via the Germplasm Resource Information Network (http://www.ars-grin.gov). The presence or absence of a pigmented testa on the grains was visually assessed (three seeds per accession) after removal of the pericarp on the dorsal side and scored as 1 and 0, respectively (File S2). Eleven accessions that showed a segregating phenotype were dropped from the analysis. Pericarp pigmentation was visually assessed for three seeds per accession, scored as 0 for white and 1 for red or yellow (File S2). Brown pericarp accessions were dropped from the analysis because this phenotype is known to be caused by the spread of tannin from the testa and masks the expression of the *R* and *Y* genes (Doggett 1988). For analysis of structuring of tannin phenotypes, we obtained testa pigmentation data (*n* = 14,785) in world sorghum collections from the Germplasm Resource Information Network. For the RIL family, phenotyping was performed in the 2007 and 2008 *kharif* (rainy) season in Patancheru, India, with presence/absence of pigmentation scored for 10-wk-old seedlings (coleoptile color) or physiologically mature plants (testa and adult plant color; Vinayan 2010).

### Genomic analysis

Genome-wide mapping in RILs and the association panels was carried out using a GLM, mixed linear model (MLM), or compressed mixed linear model (CMLM) with population parameters previously determined (Zhang *et al.* 2010) as implemented in the Genomic Association and Prediction Integrated Tool (Lipka *et al.* 2012). When a population structure (Q) term was included, the model selection feature of the Genomic Association and Prediction Integrated Tool was used to determine the optimal number of principal components (Zhu and Yu 2009). Genetic structure of the world sorghum populations was estimated by the use of principal components analysis implemented by cmdscale in R (R Core Team 2012).

Loss-of-function genome scan *P*-value for a given SNP (loss of function, *i.e.*, LOF) is calculated with binomial tests in R (R Core Team 2012) as follows:

$$\text{LOF}=\min\left(\text{binom}.\text{test}\left(\text{x}={\text{L}}_{1},\text{n}={\text{L}}_{1}\;+\;{\text{W}}_{1},\text{p}=\text{P}\right),\text{binom}.\text{test}\left(\text{x}={\text{L}}_{2},\text{n}={\text{L}}_{2}\;+\;{\text{W}}_{2},\text{p}=\text{P}\right)\right),$$

where L_1_ and L_2_ are the counts of the loss-of-function phenotype for alleles 1 and 2 (“successes”), respectively, W_1_ and W_2_ are the counts of the wild-type phenotype for alleles 1 and 2 (“failures”), respectively, and P is the overall proportion of loss-of-function phenotypes given by *P* = (L_1_+ L_2_)/(L_1_+ L_2_+ W_1_+ W_2_).

## Results

### Grain tannin GWAS in a small association panel

To identify loci underlying natural variation in grain tannin pigmentation, we first characterized associations between published tannin (presence/absence) phenotypes from a small (*n* = 142) global diversity panel (Wu *et al.* 2012) and genotypes from a 265,487 SNP genotyping-by-sequencing data set (Morris *et al.* 2013). Included in the GBS SNP map is a G-to-T transition in the *Tannin1* coding region (S4_61667908) that is 218 bp upstream of, and in perfect linkage disequilibrium with, the G-deletion that is causative for the *tan1-a* null allele (*n* = 161) (Wu *et al.* 2012). Therefore, we can use this SNP (hereafter referred to as the *tan1-a* SNP) as a positive control to compare mapping approaches. Using a simple model without statistical control for population structure (General Linear Model; GLM) we find that, as expected, the most significant association signals lies at ~61 Mb on chromosome 4 colocalized with the *Tannin1* locus (Figure S1A-B and Table 1). However, the *tan1-a* SNP is not the most significant association in this region (rank = 230; *P* < 10^−8^). Instead, the most significant association peaks are found in the ~1-Mb region surrounding *Tannin1*, at 61.1 Mb (S4_61121403; *P* < 10^−12^), 61.2 Mb (S4_61233495; *P* < 10^−10^), and 61.8 Mb (S4_61862778; *P* < 10^−12^). Including the *Tannin1* locus, we observe at least 13 peaks of association that are significant at a nominal Bonferroni-corrected *P*-value of 0.01 (*P* < 2 × 10^−7^), among which are additional candidate loci for genetic control of tannin pigmentation (File S3).

**Table 1:** Summary of testa pigmentation GWAS

SNP (Relevance)	S4_61667908 (*tan1-a* allele)	S4_61862778 (tags Tan1, -0.2 Mb)	S4_61121403 (tags Tan1, -0.5 Mb)	S4_62353772 (tags Tan1, +0.7 Mb)
Small association panel (*n* = 142)				
GLM	230 (3 × 10^−7^)	1 (4 × 10^−11^)	2 (5 × 10^−11^)	38 (8 × 10^−8^)
CMLM (K)	82 (2 × 10^−5^)	3 (6 × 10-8)	1 (2 × 10^−8^)	159,802 (0.76)
CMLM (Q + K)	82 (2 × 10^−5^)	3 (6 × 10^−8^)	1 (2 × 10^−8^)	159,802 (0.76)
MLM (K)	176 (1 × 10^−6^)	2 (7 × 10^−10^)	1 (7 × 10^−10^)	12 (8 × 10^−9^)
Large association panel (*n* = 336)				
GLM	9 (1 × 10^−14^)	3 (2 × 10^−17^)	14 (4 × 10^−15^)	1 (2 × 10^−22^)
GLM (Q)	1 (9 × 10^−15^)	3 (5 × 10^−12^)	5 (2 × 10^−10^)	14 (2 × 10^−8^)
CMLM (K)	69,491 (0.76)	77,062 (0.86)	1 (1 × 10^−11^)	61,782 (0.64)
CMLM (Q + K)	14355 (0.09)	39,506 (0.33)	1 (9 × 10^−11^)	3381 (0.01)
MLM (K)	1 (2 × 10^−13^)	115,088 (0.83)	2 (1 × 10^−11^)	87,702 (0.57)
Loss-of-function	1 (3 × 10^−19^)	4 (1 × 10^−13^)	22 (1 × 10^−9^)	26 (2 × 10^−9^)

Mapping results for various GWAS models giving the rank of the given SNP of 265,487 SNPs (*P* values in parenthesis). GWAS, genome-wide association studies; GLM, general linear model; CMLM, compressed mixed linear model; MLM, mixed linear model; SNP, single-nucleotide polymorphism.

Given the extensive population structure in sorghum, as well as kinship among elite sorghum lines, we also carried out GWAS using MLMs that take into account population structure (Q) and/or kinship (K) (Table 1). We fit both standard MLMs (Yu *et al.* 2006), which treat each individual as an independent group when fitting the random effect of kinship, and CMLMs, which have been shown to outperform GLMs and standard MLMs in some cases (Zhang *et al.* 2010). In addition, we estimated the optimal number of principal components to include as fixed effects (Q) on the basis of model selection and Bayesian information criteria (Zhu and Yu 2009). The genomic locations of GWAS peaks from the GLM are largely unchanged with the CMLM (Figure S1C-D and Table 1), although the nominal significance of the peaks is reduced. All the nominally significant association peaks identified with the GLM remain in the CMLM, although most fall below the threshold for genome-wide significance. The top associations remain near *Tannin1* on chromosome 4 but controlling for population structure and kinship with a MLM (MLM and CMLM) does not improve the mapping resolution at the *Tannin1* locus, as the *tan1-a* SNP remains less significant (*P* < 10^−4^) than several other SNPs in the ~1 Mb region surrounding *Tannin1* (*P* < 10^−6^).

### Grain tannin GWAS in a large association panel

**Figure 1:**
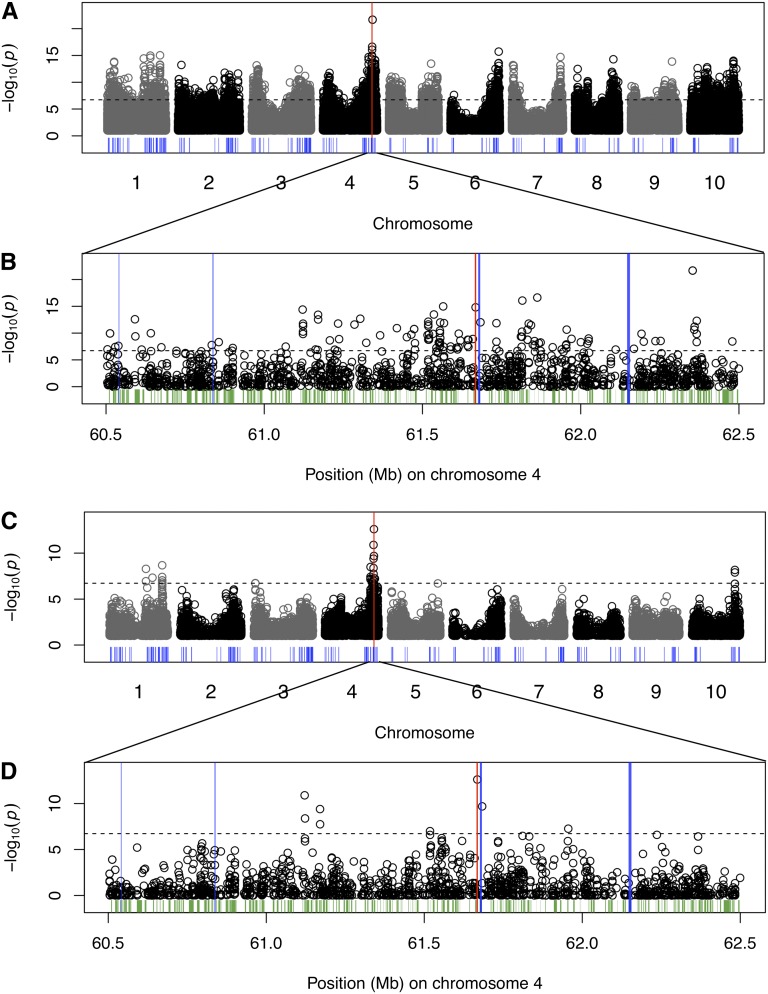
Genome-wide association study of testa presence using linear models. Manhattan plots using a GLM (A, B) and standard MLM kinship (K) (C, D) with 265,487 SNPs and 336 accessions, with (B, D) detailed view at *Tannin1* locus on chromosome 4 for GLM and MLM (K), respectively. Although the *Tannin1* locus is identified as the major effect locus for tannin presence with GLM, the *Tannin1* gene (red bar) is precisely identified with a MLM. Other flavonoid-related genes are indicated by the blue bars, whereas all other annotated genes in the detailed view are indicated in green.

If the inability to precisely map *Tannin1* in the small association panel is caused by a small sample size, then increasing the size of the association panel should improve the mapping resolution. Therefore, we phenotyped testa presence/absence in the sorghum association panel (Casa *et al.* 2008) and performed GWAS by using a variety of linear models (*n* = 336; Table 1). Fitting a GLM with this larger panel, the association for the *tan1-a* SNP is increased in rank and significance (Figure 1A-B; rank = 9; *P* < 10^−14^), but is still not the top association. Adding control for population structure and kinship using CMLM (K or Q+K), we find the rank and significance of the *tan1-a* SNP is actually reduced compared with the results using a GLM (Table 1; rank = 14355; *P* = 0.09). Finally, a GLM with population term (Q) or a standard MLM (K only; Figure 1C-D) that treats each individual as a separate group for estimating kinship does identify the *tan1-a* SNP precisely (rank = 1; *P* < 10^−15^ and *P* < 10^−12^, respectively).

**Figure 2:**
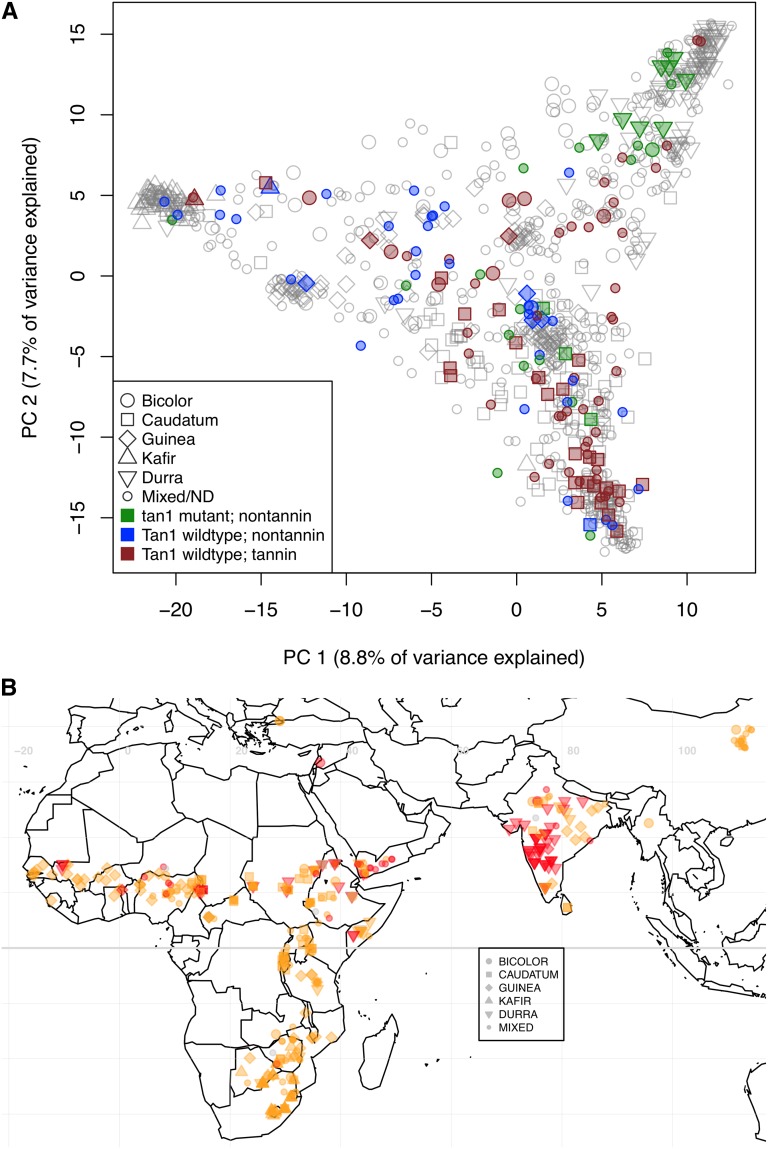
Distribution of tannin phenotype and loss-of-function alleles in a worldwide sorghum diversity panel. (A) Accessions plotted according to the first two principal components of sorghum population structure (small panel; *n* = 142), with phenotyped accessions color-coded by *Tannin1* allele and tannin phenotype (nonphenotyped accessions in gray). Although some nontannin accessions are explained by *tan1-a* or *tan1-b* (green), others must be due to additional loss-of-function alleles (blue). (ND: Not Determined) (B) Distribution of wild-type allele (G; orange) and loss-of-function allele (T; red) for the *tan1-a* SNP (S4_61667908) in source-identified sorghum accessions.

Why do some models that account for population structure (CMLM) perform worse than a naive model (GLM), generating a false-negative result for the *tan1-a* SNP? To better understand the population structure of natural variation in tannins, we characterized the distribution of pigmented testa phenotype in worldwide sorghum collections. The tannin trait segregates in all the botanical races of sorghum but shows modest population structure, with durra and guinea types having the lowest proportion of tannin accessions (15%) and caudatum and guinea-caudatum accessions having the greatest proportion (76% and 83%, respectively). However, with respect to model selection in GWAS, the structuring of the trait itself may be less important than structuring of the alleles underlying the trait. The *tan1-a* allele is found at high frequency in African and Indian durra accessions and at low frequency in Chinese and southern African accessions (Figure 2). The *tan1-a* allele explains the tannin phenotypes in all durra-derived accessions studied, but only partially accounts for the phenotype in caudatum accessions and not at all in guinea accessions. Among caudatum, guinea, and kafir types there are numerous accessions that have wild-type *Tannin1* coding regions yet have nontannin phenotypes, which suggests that the population structuring of heterogeneous alleles may account for the overcorrection by the CMLM and the effective correction by the MLM.

### Mapping of flavonoid traits in a RIL family

**Figure 3:**
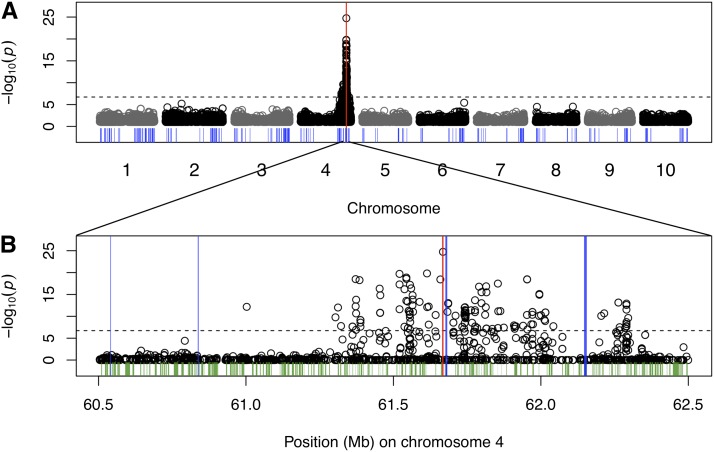
Genome-wide mapping of testa presence in a RIL biparental family. Manhattan plots for a linear model using 263 recombinant inbred lines genotyped at 265,487 SNPs, (A) scanning genome-wide and (B) with a detailed view at *Tannin1* locus on chromosome 4, with *Tannin1* indicated by the red bar. Other flavonoid-related genes are indicated by the blue bars, whereas all other annotated genes in the detailed view are indicated in green.

Given that complex association signals made the precise mapping of *tan1-a* in an association population difficult, we considered whether the reduced genetic and allelic heterogeneity of a biparental family would allow precise mapping of *Tannin1* using linear models. We phenotyped testa pigmentation (tannin presence) in a population of 263 RILs that we genotyped at 265,487 SNPs. There is a single locus associated with the pigmented testa in this family that is precisely colocalized with *Tannin1* on chromosome 4, with the most significant SNP being the *tan1-a* SNP (Figure 3). To determine whether this gene resolution mapping in RILs is likely to be a typical result, we also mapped two other flavonoid pigmentation traits (coleoptile color and adult plant color) that are segregating in this RIL family. We mapped coleoptile color to a region around 54 Mb on chromosome 6 (Figure S2). This peak colocalizes with the classical *Rs1* locus and *a priori* candidate gene *Sb06g025060*, a putative basic helix-loop-helix (bHLH) transcription factor, and a sorghum co-ortholog of Arabidopsis *TRANSPARENT TESTA8* and maize *B1*/*R1* anthocyanin regulators (File S3). However, the most significant SNP (S6_53849573) is 220 kb upstream of *Sb06g025060*, and no promising *a posteriori* candidate genes are found at S6_53849573, suggesting gene resolution was not achieved in this case. Adult plant color maps to 58 Mb on chromosome 6, colocalized with the classical *P* locus (Doggett 1988; Mace and Jordan 2010) and a large cluster of putative reductase genes that are homologous to Arabidopsis *TRANSPARENT TESTA3* and *BANYULS* and maize *ANTHOCYANINLESS1* (File S1). Here again, the most significant SNP (S6_57865283) is not colocalized precisely with the *a priori* candidates (*TT3*/*BANYULS* cluster) but 260 kb upstream (Figure S3).

### Loss-of-function genome scan on grain tannin

**Figure 4:**
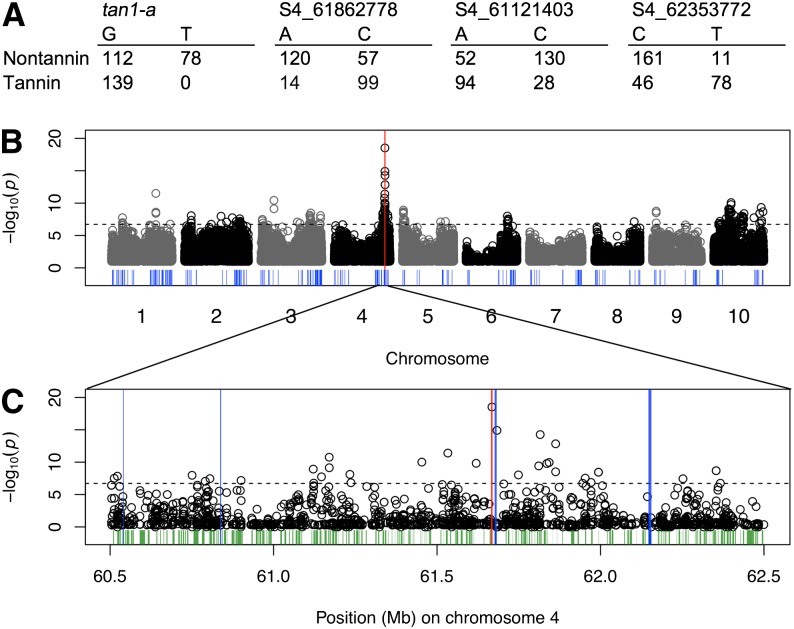
Genome-wide mapping of testa presence using a loss-of-function genome scan. (A) Allele counts for the *tan1-a* SNP and other SNPs near *Tannin1* that are associated with a nontannin phenotype. The nominal *P*-value is from a binomial test on the putative loss-of-function allele at each SNP, (B) scanning genome-wide, and (C) a detailed view of the *Tannin1* locus on chromosome 4, with *Tannin1* indicated by the red bar. Other flavonoid-related genes are indicated by the blue bars, whereas all other annotated genes in the detailed view are indicated in green.

Why did several of the linear models we tested fail to precisely identify the *tan1-a* allele in association panels, even though it is common and highly penetrant? A comparison of the 2 × 2 contingency tables for the *tan1-a* SNP *vs.* the more significant SNPs provides some insight here (Figure 4A). The loss-of-function *tan1-a* allele shows striking covariation with the testa phenotype (T allele: nontannin = 78 *vs.* tannin = 0) but little signal of covariation for the wild-type allele (G allele: nontannin = 112 *vs.* tannin = 139). Although this lack of covariation for the wild-type allele is to be expected (because there is no reason that accessions carrying the wild-type allele at *Tannin1* cannot carry loss-of-function alleles at other loci), it reduces the significance of a linear model fitting the genotype-phenotype association. In contrast, the other more significant SNPs near *Tannin1* show covariation for both alleles (in opposite directions), with the wild-type allele more often found with the wild-type phenotype (Figure 4A). This pattern of covariation increases the significance of the fit of a linear model or contingency test, even though the genotype-phenotype covariation for the wild-type allele is irrelevant when considering a loss-of-function polymorphism.

To investigate an approach that may be appropriate for loss-of-function alleles we used a simple heuristic genome scan based on a binomial test (see the section *Materials and Methods*). We identified SNPs with alleles that are often found in individuals with the loss-of-function phenotype (testa absent) and rarely or never found in individuals with the wild-type phenotype (testa present). In effect, the phenotypes at the putative wild-type allele at each SNP is ignored. With this loss-of-function genome scan approach, the *tan1-a* SNP is precisely identified whereas other SNPs near the *Tannin1* locus that had strong indirect associations with linear modeling have reduced association signals (Figure 4, B and C, Table 1, and Figure S4). Thus, in this case we find that a simple heuristic scan that considers the underlying genetics of the trait outperforms more sophisticated models.

### Pericarp pigmentation GWAS

**Figure 5:**
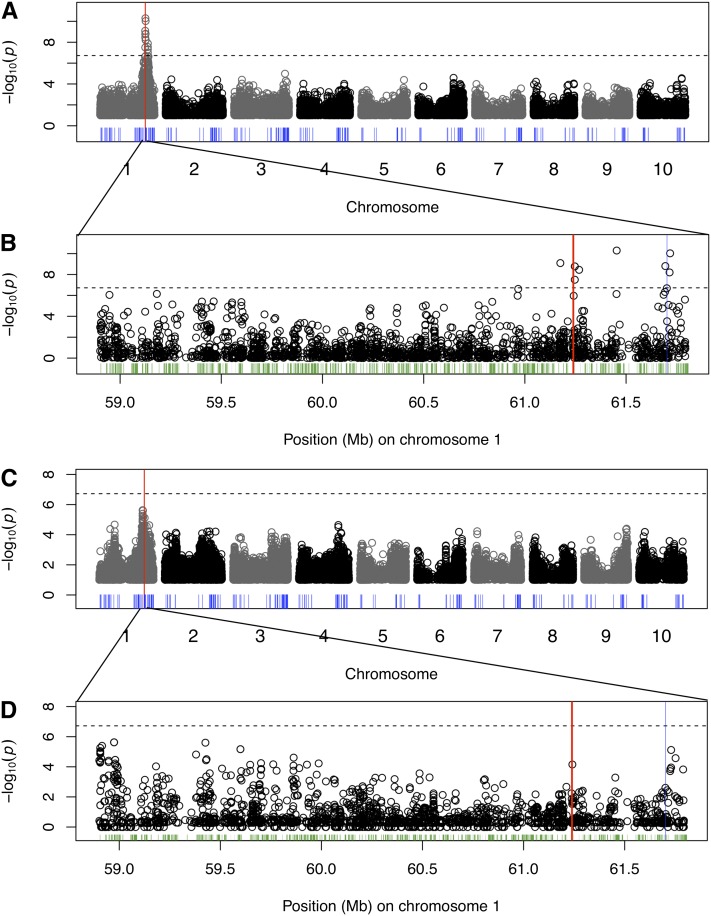
GWAS of white pericarp phenotype. Manhattan plots for GWAS using CMLM kinship (K) (A, B) and a loss-of-function genome scan (C, D) and with 265,000 SNPs and 347 accessions, showing a (A, C) genome-wide and (B, D) a detailed view around *Y1* on chromosome 1, with the *Y1* gene indicated by the red bar. Other flavonoid-related genes are indicated by the blue bars, whereas all other annotated genes in the detailed view are indicated in green. ×

Although we found that gene-resolution mapping was possible with *Tannin1*, we wondered whether this would be true for other flavonoid pigmentation traits. To assess the mapping resolution in this panel with another trait, we used the white pericarp phenotype, which reflects a lack of flavonoid pigmentation in the outer seed coat (Doggett 1988; Ibraheem *et al.* 2010). It is known that white pericarp phenotype can be caused by loss-of-function mutations in the *Yellow seed1* gene (*Y1*, Chr1: 61,237,360–61,241,520; Ibraheem *et al.* 2010). Note, the *Y1* gene was cloned based on the excision of a transposable element, not genetic analysis of natural variation, so we do not have a validated genetic variant of *Y1* in the SNP data as we did for the *Tannin1* case. However, it is known from the classical inheritance literature that natural variation at the *Y* locus exists (Rooney 2000). As expected, the most significant associations were found around 61 Mb on chromosome 1 (Figure 5). As was the case for *Tannin1*, though, the *Y1* gene was broadly but not precisely identified: among the top associations are SNPs that are flanking *Y1* (5 kb from *Y1*, S1_61246791; *P* < 10^−8^) but the most significant association are ~0.5 Mb from *Y1* (S1_61717652). Because white pericarp represents loss-of-function phenotype, we also used the loss-of-function genome scan approach for the pericarp pigmentation trait. In this case, the loss-of-function genome scan does not improve the mapping resolution as compared to the linear models.

## Discussion

### Genetics of flavonoid pigmentation

GWAS have been useful to characterize the contribution of known and novel flavonoid pigmentation genes in several plant species (Atwell *et al.* 2010; Huang *et al.* 2010; Cockram *et al.* 2010). Given that control of flavonoids by a WD40-bHLH-MYB regulatory system is broadly conserved (Petroni and Tonelli 2011), it seems likely that these regulators underlie some natural variation in sorghum pigmentation. Is there evidence of natural variation in each of the three types of transcription factors that control tannins in plants (WD40, bHLH, MYB)? From classical inheritance studies, it is known that at least two loci control the presence of brown coloration in grain subcoat (*B1* and *B2*) that have been mapped to chromosome 2 and chromosome 4, and one of these was cloned as WD40 gene *Tannin1* (Rami *et al.* 1998; Mace and Jordan 2010; Wu *et al.* 2012). On the basis of a comparison of *B1*/*B2* genotypes from classical inheritance studies (Doggett 1988; Rooney 2000) and *Tannin1* genotypes (Wu *et al.* 2012), it can be inferred that *Tannin1* corresponds to *B2*. Multiple studies have identified a second major effect locus controlling tannin presence at around 8 Mb on chromosome 2, and from the *Tannin1*-controlled GWAS (File S3), there is evidence that the gene underlying *B1* is the putative bHLH transcription factor *Sb02g006390*. This study is based on qualitative tannin phenotypes (presence/absence) so quantitative phenotyping may reveal additional loci underlying the observed variation among intermediate and high tannin varieties (Gu *et al.* 2004).

Given that flavonoid-related gene families are well-characterized and well-conserved across distant plant lineages, we would expect that most association peaks would colocalize to *a priori* candidate genes. Although a number of peaks do colocalize precisely with candidate genes, many more of them do not, despite the large candidate gene set and the liberal inclusion criteria. It is possible that some unexpected signals represent true associations at novel genes, though it is unlikely for most peaks. In some cases, these unexpected signals may represent stochastic noise, spurious associations that do not reflect any underlying genetic heritability. However, most of these peaks increased in significance with a larger sample size so they are unlikely to represent stochastic noise and more likely to represent indirect associations (Platt *et al.* 2010). In particular, the significant SNPs near *Tannin1* are likely to represent synthetic associations (Goldstein 2011), instances in which a phenotype caused by multiple rare alleles is spuriously assigned to a common allele that is linked.

### Mapping loss-of-function alleles

The development of experimental design strategies and statistical methods that account for complex genetic architecture and population structure in genome-wide mapping studies is an active area of research (Zhang *et al.* 2010; Zhao *et al.* 2011; Huang *et al.* 2012; Lipka *et al.* 2012; Segura *et al.* 2012). Here we were able to compare several mapping approaches empirically by using the testa presence/absence phenotype in sorghum and a GBS SNP map that includes a validated SNP in the *Tannin1* major effect gene. All the approaches we tested here identified the *Tannin1* locus, broadly defined (+/− 1 Mb of the *Tannin1* gene), as the major locus underlying testa presence/absence, but the precise identification of the *Tannin1* gene was not consistently obtained. Increasing the panel size increased the significance of the *tan1-a* SNP relative to the indirect associations. Given that minor allele frequencies (MAFs) of indirect associations (*i.e.*, the SNPs near *Tannin1* that have greater association signals than the *tan1-a* SNP) are greater (MAF = 0.3 – 0.45) than the *tan1-a* SNP (MAF = 0.2), these indirect associations fit the expectation for synthetic associations: older, more widespread SNPs that tag a haplotype block on which multiple loss-of-function alleles of *Tannin1* have arisen (Dickson *et al.* 2010; Orozco *et al.* 2010). Although methods have been developed to account for epistasis by stepwise model fitting (Segura *et al.* 2012), it will be difficult to derive benefits from these models if the most significant association in the first model step is itself an indirect association. Given that allelic heterogeneity is abundant among the handful of well-characterized sorghum genes [*Tannin1* (Wu *et al.* 2012), *Maturity1* (Murphy *et al.* 2011), and *Shattering1* (Lin *et al.* 2012)] synthetic associations could be a common feature of major effect loci in sorghum and other species of similar population structure.

Loss-of-function variants are a common source of natural variation, and recently methods have been developed to identify multiple, low-frequency loss-of-function alleles at the same gene in GWAS (Liu *et al.* 2011). The loss-of-function genome scan approach we used here allowed us to precisely map a high frequency loss-of-function allele to the exclusion of nearby indirect associations. Note, that although we applied this approach to binary phenotypes the principle does not depend on binary phenotypes. Rather, it depends on the epistasis that exists because the loss-of-function allele cannot be rescued (*i.e.*, wild-type phenotype is never found with loss-of-function allele) while the wild-type allele can easily be found in an individual that harbors an independent loss-of-function allele at a different locus (*i.e.*, loss-of-function phenotype is often found with wild-type allele).

### Improving resolution with genomic data

We found that mapping in a biparental family using high-density SNP markers can achieve gene resolution. Traditionally, high-resolution SNP maps have not been used for biparental families due to technical limitations and the expectation that the small number of recombination events limits the utility of greater marker density. The gene-resolution mapping of *Tannin1* in a modest-sized (*n* = 263) RIL family with simple phenotyping (field-based scoring of testa presence/absence) demonstrates that the combination of advanced mapping populations with high-resolution genotyping is an effective strategy for trait dissection (Bergelson and Roux 2010; Brachi *et al.* 2011). Still, the lower-resolution mapping results for the adult plant color and coleoptile color traits in the RILs suggest that consistent gene-level mapping will require larger families and/or advanced multi-parent mapping approaches (Jordan *et al.* 2011). Given the substantial investment that has already been made in RIL development, the cost-effectiveness of GBS, and the suitability of the system for “genotype once, phenotype many times” approach, a broader effort to make high-density genotypes available for existing advanced mapping populations seems warranted.

It is worth noting that if the *tan1-a* SNP had not, by chance, been represented in the GBS SNP map, the results of the loss-of-function genome scan would have been qualitatively equivalent to the linear models. This highlights that to derive a benefit from the loss-of-function genome scan approach over linear models the density of genotyping must be high enough that causative SNPs, or SNPs in perfect LD with the causative polymorphism (*e.g.*, *tan1-a*), are represented in the genotyping data. A lack of a causative or perfectly linked SNP for pericarp pigmentation would explain why both the linear model and loss-of-function genome scan approaches achieved just Mb resolution for this trait. Similarly in the RIL mapping, had the *tan1-a* SNP not been represented the other SNPs in the data set would not have precisely tagged the *Tannin1* gene. Although the identification of *a priori* candidates through comparative genomics provides some guidance, there are typically many reasonable candidates within Mb-scale mapping intervals; for example, around 60−63 Mb on chromosome 4, there are four other homologs of tannin genes that would have been equally promising candidates. As genomic coverage increases, imputation methods improve, and mapping populations are refined and expanded, genome-wide mapping approaches will increasingly need to be optimized to identify causative variants as opposed to tagging SNPs (Huang *et al.* 2012). Given the ever-lowering costs of sequencing *vs.* the high cost of candidate gene validation efforts, the use of whole-genome resequencing to increase the resolution of mapping studies is likely to be cost effective. Although this study of relatively simple pigmentation traits highlights the trade-offs between population- and family-based mapping approaches because of the complexity of association signals, in either approach high density genotyping can facilitate gene resolution mapping of traits.

## Supplementary Material

Supporting Information
