# Supplementary material for: Dissecting Genome-Wide Association Signals for Loss-of-Function Phenotypes in Sorghum Flavonoid Pigmentation Traits
Source: G3 (Bethesda). 2013 Nov 1;3(11):2085–94. doi: 10.1534/g3.113.008417 (PMC3815067; doi:10.1534/g3.113.008417)
Supplement: Supporting Information [file supp_3_11_2085__index.html]

Dissecting Genome-Wide Association Signals for Loss-of-Function Phenotypes in Sorghum Flavonoid Pigmentation Traits — Supporting Information 

# Dissecting Genome-Wide Association Signals for Loss-of-Function Phenotypes in Sorghum Flavonoid Pigmentation Traits

## Supporting Information for Morris *et al.*, 2013

**Files in this Data Supplement:**

- Supporting Information - Figures S1-S4, Table S1, and Files S1-S3 (PDF, 733 KB)
- Figure S1 - Genome-wide association study of tannin presence in a small association panel. (PDF, 494 KB)
- Figure S2 - Genome-wide association mapping of coleoptile color in a RIL family. (PDF, 381 KB)
- Figure S3 - Genome-wide association mapping of adult plant color in a RIL family. (PDF, 392 KB)
- Figure S4 - Distribution of test statistic for the loss-of-function genome scan. (PDF, 348 KB)
- Table S1 - Summary of flavonoid-related candidate gene families in sorghum. (PDF, 322 KB)
- File S3 - Supporting Discussion (PDF, 318 KB)
- File S1 - Complete listing of 365 *a priori* candidate genes in flavonoid-related gene families. (.xlsx, 40 KB)
- File S2 - Flavonoid pigmentation phenotypes for association panel and recombinant inbred lines. (.xlsx, 57 KB)
