# Supplementary material for: Dissecting Genome-Wide Association Signals for Loss-of-Function Phenotypes in Sorghum Flavonoid Pigmentation Traits
Source: G3 (Bethesda). 2013 Nov 1;3(11):2085–94. doi: 10.1534/g3.113.008417 (PMC3815067; doi:10.1534/g3.113.008417)
Supplement: Supporting Information [file supp_g3.113.008417_008417SI.pdf]

G3 · Genes | Genomes | Genetics

Dissecting genome-wide association signals for loss-of-function phenotypes in sorghum flavonoid pigmentation traits

Geoffrey P. Morris[*,1], Davina H. Rhodes[*], Zachary Brenton[*], Punna Ramu[§], Vinayan Madhumal Thayil[§,2], Santosh Deshpande[§], C. Thomas Hash[†], Charlotte Acharya[‡], Sharon E. Mitchell[‡], Edward S. Buckler[**], Jianming Yu[§§], and Stephen Kresovich[*]

[*] Department of Biological Sciences, University of South Carolina, Columbia, SC 29208, USA, [§] ICRISAT, Patancheru PO, Hyderabad 502 324, Andhra Pradesh, India, [†]ICRISAT-Sadoré, BP 12404, Niamey, Niger, [‡] Institute for Genomic Diversity, Cornell University, Ithaca, NY 14853, USA, [**] USDA-ARS, Department of Plant Breeding and Genetics, Cornell University, Ithaca, NY 14853, USA, [§§] Department of Agronomy, Iowa State University, Ames, Iowa 50011, USA.

[1] Corresponding author: Department of Biological Sciences, University of South Carolina, Columbia, SC 29208, USA. E-mail: morrisgp@mailbox.sc.edu

DOI: 10.1534/g3.113.008417

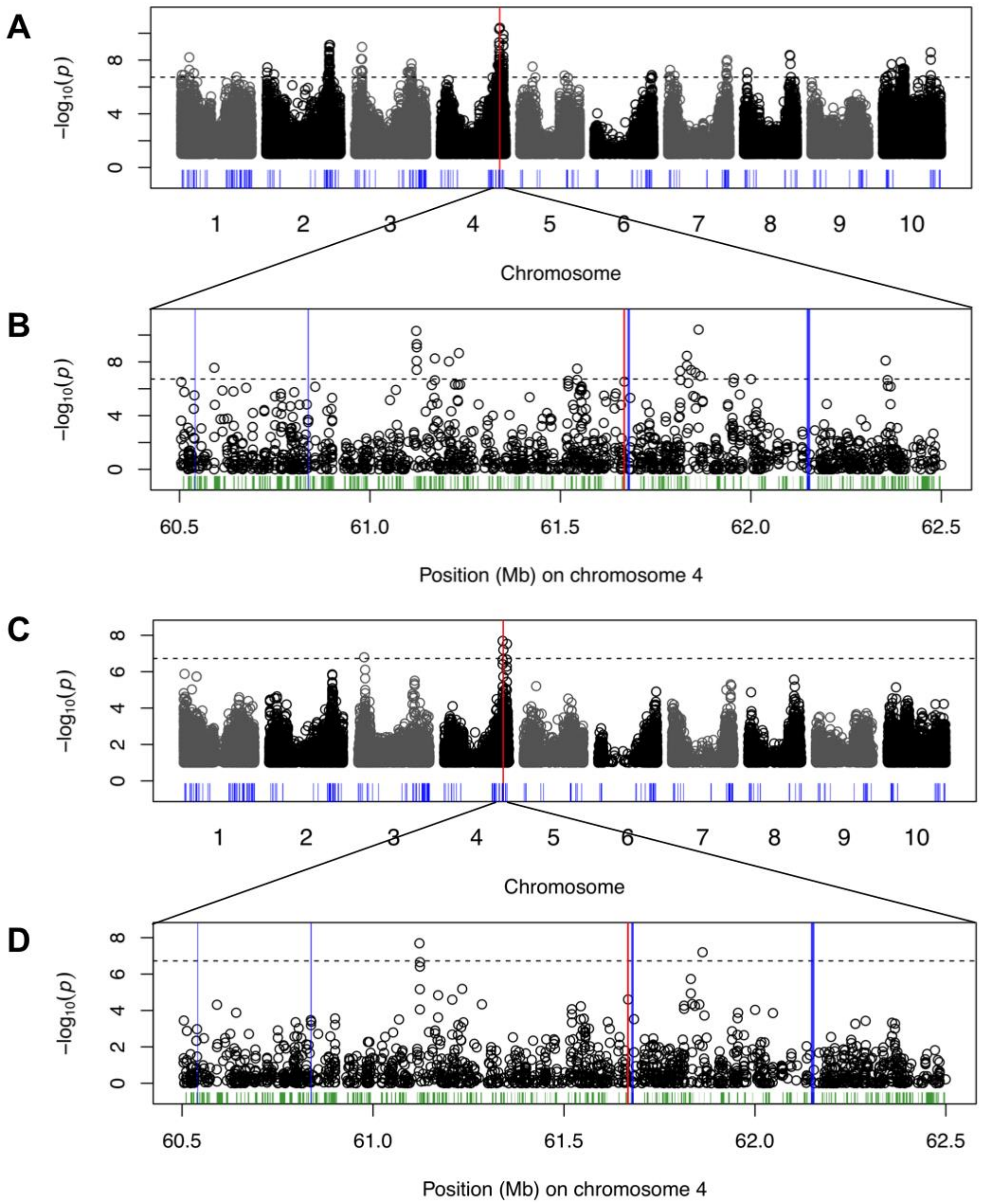

Figure S1 Genome-wide association study of tannin presence in a small association panel. Manhattan plots for GWAS using a (A,B) general linear model (GLM) and (C,D) compressed mixed linear model (MLM [K]) with 265,487 SNPs and 142 accessions. The horizontal line indicates the Bonferroni significance threshold at a 5% nominal family-wise error rate. The *Tannin1* locus is identified with Mb resolution but the *Tannin1* gene (red bar) is not precisely identified with either model. Other flavonoid-related genes are indicated by the blue bars, while all other annotated genes in the detailed view are indicated in green.

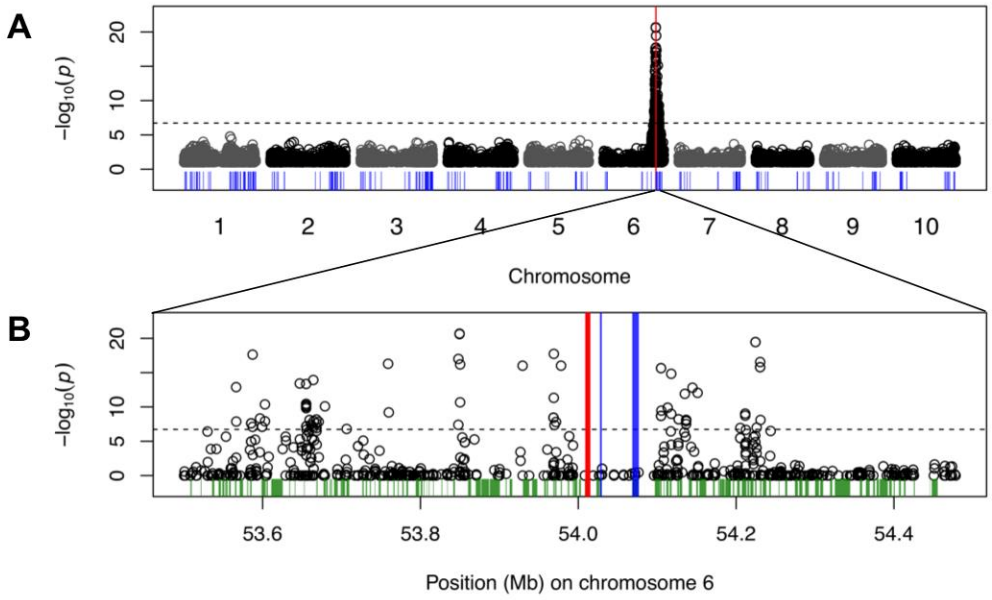

Figure S2 Genome-wide association mapping of coleoptile color in a RIL family. (A) A genome-wide Manhattan plot and (B) detailed view on chromosome 6 with a candidate gene, *SbTT8*/*Sb06g025060,* indicated by the red bar. Other flavonoid-related genes are indicated by the blue bars, while all other annotated genes in the detailed view are indicated in green.

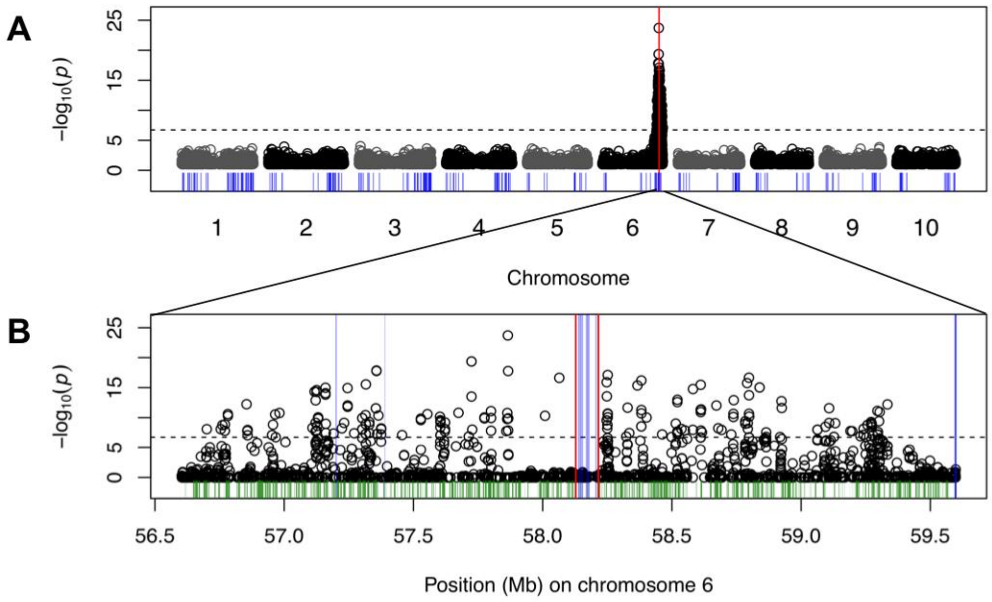

Figure S3 Genome-wide association mapping of adult plant color in a RIL family. (A) A genome-wide Manhattan plot and (B) detailed view on chromosome 6 with a cluster of candidate genes homologous to *TRANSPARENT TESTA3* and *BANYULS* indicated by the red bars. Other flavonoid-related genes are indicated by the blue bars, while all other annotated genes in the detailed view are indicated in green.

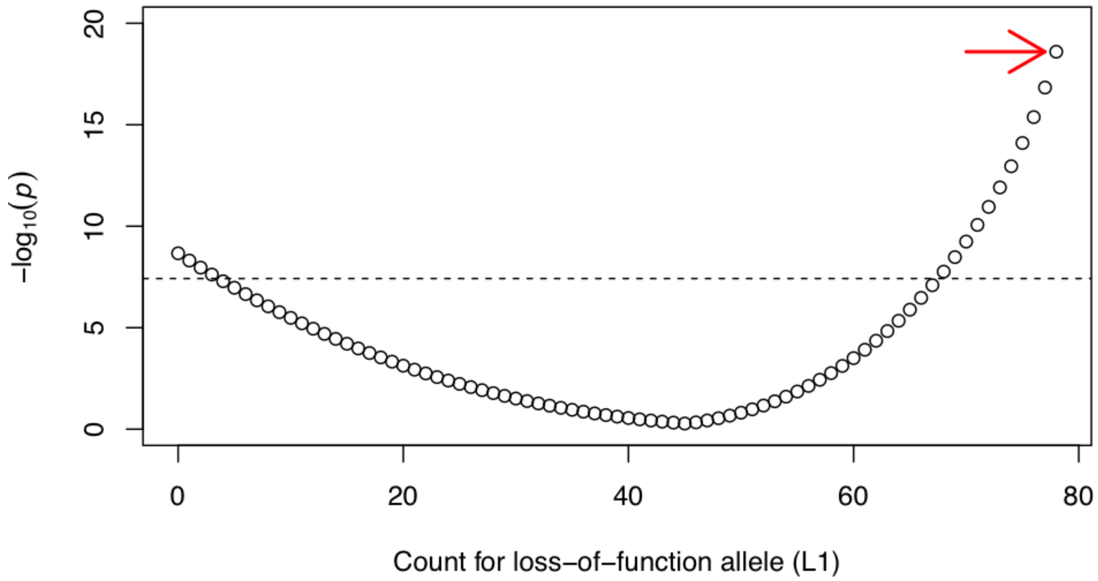

Figure S4 Distribution of test statistic for the loss-of-function genome scan. Plotted are the negative $\log_{10}$ *P*-values for every 2×2 contingency table with the same marginal frequencies as the observed contingency table for the *tan1-a* allele (*i.e.* same overall frequencies of phenotypes and genotypes). The dashed line indicates the Bonferroni-corrected *P*-value of 0.01 and the red arrow indicates the observed test statistic for the *tan1-a* allele.

Files S1-S2

Available for download at http://www.g3journal.org/lookup/suppl/doi:10.1534/g3.113.008417/-/DC1

File S1

Complete listing of 365 *a priori* candidate genes in flavonoid-related gene families.

File S2

Flavonoid pigmentation phenotypes for association panel and recombinant inbred lines

File S3

SUPPORTING DISCUSSION

Evidence for other tannin loci from small association panel GLM: The secondary peak from the GLM is found at around 66-67 Mb of chromosome 2, with top SNPs at 66.7 Mb (S2_66783467; $p < 10^{-9}$) and 66.0 Mb (S2_66003858; $p < 10^{-9}$). This diffuse association peak colocalizes with several *a priori* candidate genes (Supporting table 2), including putative MYB transcription factor *Sb02g031190*, which is the sorghum ortholog of maize flavonoid regulator *ZmMYB31* (Fornalé *et al.* 2010). With the CMLM the only association peak outside the *Tannin1* region that is significant at the genome-wide threshold is at 6.2 Mb on chromosome 3, which is over one Mb from the nearest *a priori* candidate gene.

To reduce the effects of genetic heterogeneity in the experiment, we controlled for known functional variants in the *Tannin1* gene by (1) including the presence of the non-functional *tan1-a* or *tan1-b* allele as a covariate in the model ("*tan1*-covariate GLM") or (2) carrying out the GWAS using only lines with *Tan1* wildtype alleles in the coding region ("*Tan1*-only GLM"; n = 112). With both approaches, the most significant association peak lie on chromosome 2 between 8.075 Mb and 8.45 Mb. This region is also present in the GLM but falls below the threshold for genome-wide significance. An *a priori* candidate gene *Sb02g006390* (Chr. 2: 8,003,227 - 8,008,714) lies at the edge of, but not directly under, the association peak. *Sb02g006390* is a putative bHLH transcription factor and a co-ortholog of two genes known to control grain tannins, rice *Rc* (Furukawa *et al.* 2007) and Arabidopsis *TRANSPARENT TESTA8* (Nesi *et al.* 2000). This association peak also colocalizes with previously described pigmented testa QTL (Mace and Jordan 2010; Wu *et al.* 2012).

A number of other significant associations are observed using the *Tan1*-only GLM. The second most significant association is at 57.9 Mb on chromosome 3 (S3_57899793; $P = 2\times10^{-8}$). The closest *a priori* candidate is a putative leucoanthocyanin reductase (LAR) found 170kb downstream. The next most significant peak spans 1.16 Mb to 1.23 Mb on chromosome 1 (top SNP: S1_1232724; $P = 4\times10^{-8}$). Within this peak (and 53kb from the top SNP) is *a priori* candidate gene *Sb01g001230*, a putative Glutathione-S-Transferase and sorghum ortholog of Arabidopsis *TRANSPARENT TESTA19* (Kitamura *et al.* 2004). Finally, even after controlling for known *Tannin1* loss-of-function alleles (*tan1-a* and *tan1-b*) there are still marginally significant association peaks near the *Tannin1* locus (*e.g.* S4_60641625) suggesting that additional loss-of-function alleles for *Tannin1* may exist.

References

Fornalé, S., X. Shi, C. Chai, A. Encina, S. Irar *et al.*, 2010 ZmMYB31 directly represses maize lignin genes and redirects the phenylpropanoid metabolic flux. The Plant Journal 64: 633–644.

Furukawa, T., M. Maekawa, T. Oki, I. Suda, S. Iida *et al.*, 2007 The Rc and Rd genes are involved in proanthocyanidin synthesis in rice pericarp. The Plant Journal 49: 91–102.

Kitamura, S., N. Shikazono, and A. Tanaka, 2004 TRANSPARENT TESTA 19 is involved in the accumulation of both anthocyanins and proanthocyanidins in Arabidopsis. The Plant Journal 37: 104–114.

Nesi, N., I. Debeaujon, C. Jond, G. Pelletier, M. Caboche *et al.*, 2000 The TT8 Gene Encodes a Basic Helix-Loop-Helix Domain Protein Required for Expression of DFR and BAN Genes in Arabidopsis Siliques. The Plant Cell Online 12: 1863–1878.

Table S1 Summary of flavonoid-related candidate gene families in sorghum. Known components of the flavonoid network include (1) biosynthetic enzymes that build the polyphenol backbone and produce diverse end products, (2) regulators, particularly a ternary complex of WD40, MYB, and bHLH transcription factors, and (3) transport and polymerization proteins responsible for localization and stabilization of the end products.

Reference gene name	Reference gene systematic ID	Reference species	Max. % similarity	Min. % similarity	Number of genes	Functional category	Function of reference gene
TT4	AT5G13930	*A. thaliana*	91.6	12.4	44	Biosynthesis	Chalcone synthase (CHS)
TT5	AT3G55120	*A. thaliana*	56.1	35.4	2	Biosynthesis	Chalcone isomerase (CHI)
TT6	AT3G51240	*A. thaliana*	76.8	36.9	15	Biosynthesis	Flavone 3-hydroxylase (F3H)
TT7	AT5G07990	*A. thaliana*	69.8	46.4	21	Biosynthesis	Flavone 3'-hydroxylase (F3'H)
TT3	AT5G42800	*A. thaliana*	68.8	47.4	18	Biosynthesis	Dihydroflavonol reductase (DFR)
TT18/TDS4	AT4G22880	*A. thaliana*	63.8	43.5	12	Biosynthesis	Anthocyanidin synthase (ANS/LDOX)
TT15	AT1G43620	*A. thaliana*	68.6	9.1	5	Biosynthesis	UDP-flavonoid glucosyl transferase (UFGT)
Banyuls (BAN)	AT1G61720	*A. thaliana*	63.5	36.2	22	Biosynthesis	Anthocyanidin reductase (ANR)
TT10	AT5G48100	*A. thaliana*	61.2	55	14	Biosynthesis	Flavonoid oxidase
VvLAR1	GSVIVG01011958001	*Vitis vinifera*	52.9	43.9	8	Biosynthesis	Leucoanthocyanin reductase (LAR)
TT2	AT5G35550	*A. thaliana*	36.8	32.2	8	Regulation	MYB transcription factor
TT8	AT4G09820	*A. thaliana*	56	8.5	17	Regulation	bHLH transcription factor
TTG1	AT5G24520	*A. thaliana*	73	22.3	13	Regulation	WD40 repeat protein
TTG2	AT2G37260	*A. thaliana*	35.4	12.1	12	Regulation	WRKY transcription factor
TT16	AT5G23260	*A. thaliana*	53.6	36.5	17	Regulation	MADS-box transcription factor
TT1	AT1G34790	*A. thaliana*	46.9	22.1	22	Regulation	Zn-finger transcription factor
TT12	AT3G59030	*A. thaliana*	60.6	50.9	16	Transport	MATE vacuolar transport
TT19	AT5G17220	*A. thaliana*	60.7	28	24	Transport	Glutathione-S-transferase
aha10	AT1G17260	*A. thaliana*	82.5	28.9	20	Transport	H+-ATPase proton pump
Bronze2 (BZ2)	GRMZM2G016241	*Zea mays*	79.5	39.8	44	Transport	Glutathione-S-transferase
ZmMRP3	GRMZM2G111903	*Zea mays*	89.4	48.6	11	Transport	MRP anthocyanin transporter
