# Supplementary material for: Dissecting Genome-Wide Association Signals for Loss-of-Function Phenotypes in Sorghum Flavonoid Pigmentation Traits
Source: G3 (Bethesda). 2013 Nov 1;3(11):2085–94. doi: 10.1534/g3.113.008417 (PMC3815067; doi:10.1534/g3.113.008417)
Supplement: Supporting Information [file supp_g3.113.008417_FigureS1.pdf]

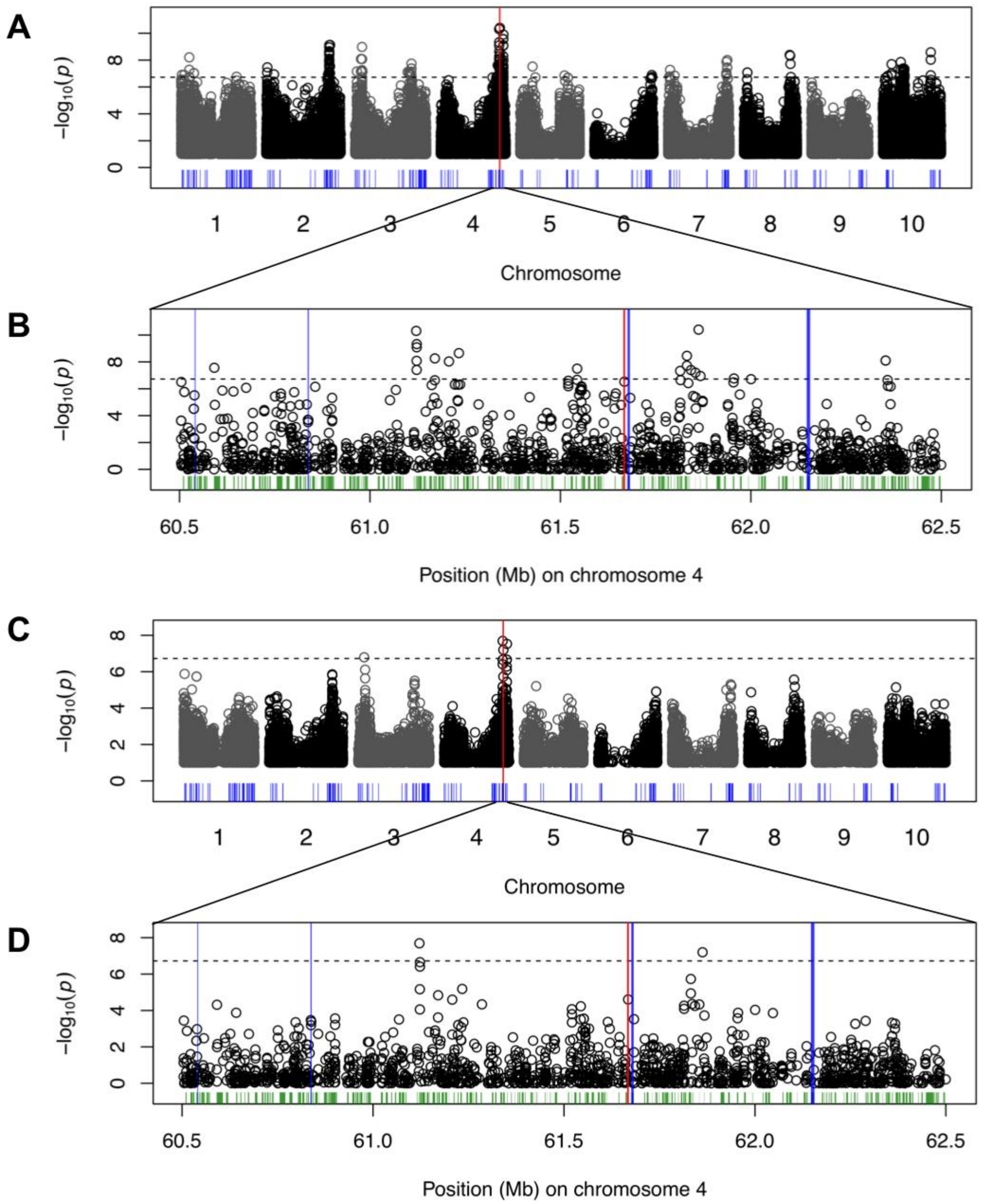

Figure S1 Genome-wide association study of tannin presence in a small association panel. Manhattan plots for GWAS using a (A,B) general linear model (GLM) and (C,D) compressed mixed linear model (MLM [K]) with 265,487 SNPs and 142 accessions. The horizontal line indicates the Bonferroni significance threshold at a 5% nominal family-wise error rate. The *Tannin1* locus is identified with Mb resolution but the *Tannin1* gene (red bar) is not precisely identified with either model. Other flavonoid-related genes are indicated by the blue bars, while all other annotated genes in the detailed view are indicated in green.
