# Supplementary material for: Dissecting Genome-Wide Association Signals for Loss-of-Function Phenotypes in Sorghum Flavonoid Pigmentation Traits
Source: G3 (Bethesda). 2013 Nov 1;3(11):2085–94. doi: 10.1534/g3.113.008417 (PMC3815067; doi:10.1534/g3.113.008417)
Supplement: Supporting Information [file supp_g3.113.008417_FigureS3.pdf]

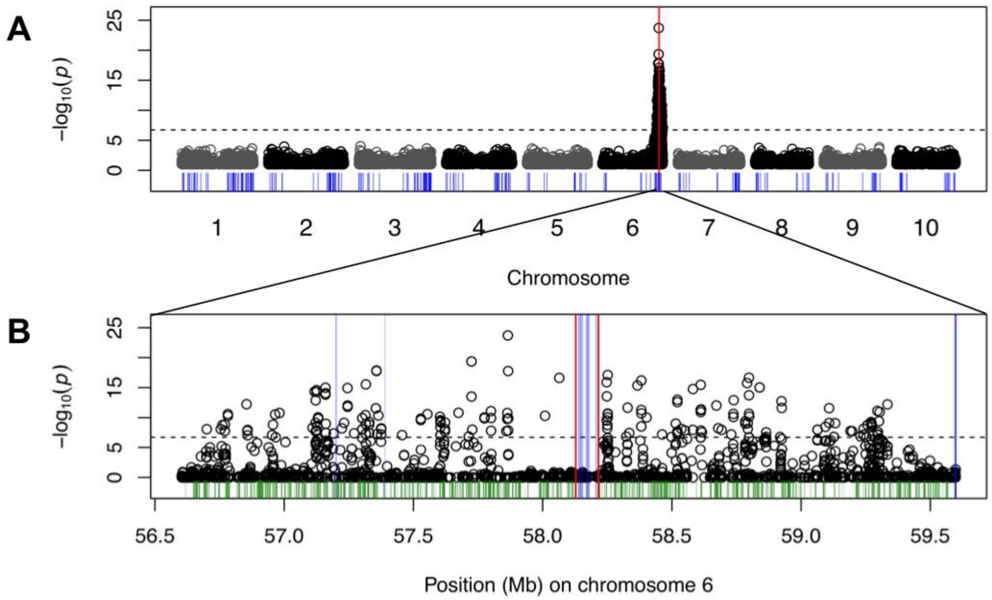

Figure S3 Genome-wide association mapping of adult plant color in a RIL family. (A) A genome-wide Manhattan plot and (B) detailed view on chromosome 6 with a cluster of candidate genes homologous to *TRANSPARENT TESTA3* and *BANYULS* indicated by the red bars. Other flavonoid-related genes are indicated by the blue bars, while all other annotated genes in the detailed view are indicated in green.
