# Supplementary material for: Dissecting Genome-Wide Association Signals for Loss-of-Function Phenotypes in Sorghum Flavonoid Pigmentation Traits
Source: G3 (Bethesda). 2013 Nov 1;3(11):2085–94. doi: 10.1534/g3.113.008417 (PMC3815067; doi:10.1534/g3.113.008417)
Supplement: Supporting Information [file supp_g3.113.008417_FigureS4.pdf]

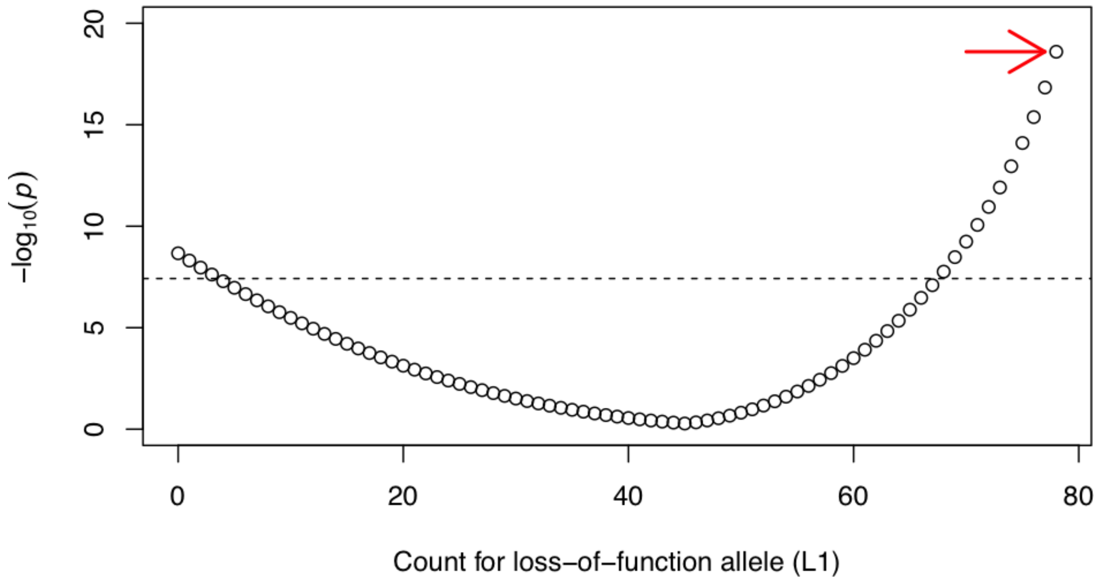

Figure S4 Distribution of test statistic for the loss-of-function genome scan. Plotted are the negative $\log_{10}$ *P*-values for every 2×2 contingency table with the same marginal frequencies as the observed contingency table for the *tan1-a* allele (*i.e.* same overall frequencies of phenotypes and genotypes). The dashed line indicates the Bonferroni-corrected *P*-value of 0.01 and the red arrow indicates the observed test statistic for the *tan1-a* allele.
