# Supplementary material for: Dissecting Genome-Wide Association Signals for Loss-of-Function Phenotypes in Sorghum Flavonoid Pigmentation Traits
Source: G3 (Bethesda). 2013 Nov 1;3(11):2085–94. doi: 10.1534/g3.113.008417 (PMC3815067; doi:10.1534/g3.113.008417)
Supplement: Supporting Information [file supp_g3.113.008417_TableS1.pdf]

Table S1 Summary of flavonoid-related candidate gene families in sorghum. Known components of the flavonoid network include (1) biosynthetic enzymes that build the polyphenol backbone and produce diverse end products, (2) regulators, particularly a ternary complex of WD40, MYB, and bHLH transcription factors, and (3) transport and polymerization proteins responsible for localization and stabilization of the end products.

Reference gene name	Reference gene systematic ID	Reference species	Max. % similarity	Min. % similarity	Number of genes	Functional category	Function of reference gene
TT4	AT5G13930	*A. thaliana*	91.6	12.4	44	Biosynthesis	Chalcone synthase (CHS)
TT5	AT3G55120	*A. thaliana*	56.1	35.4	2	Biosynthesis	Chalcone isomerase (CHI)
TT6	AT3G51240	*A. thaliana*	76.8	36.9	15	Biosynthesis	Flavone 3-hydroxylase (F3H)
TT7	AT5G07990	*A. thaliana*	69.8	46.4	21	Biosynthesis	Flavone 3'-hydroxylase (F3'H)
TT3	AT5G42800	*A. thaliana*	68.8	47.4	18	Biosynthesis	Dihydroflavonol reductase (DFR)
TT18/TDS4	AT4G22880	*A. thaliana*	63.8	43.5	12	Biosynthesis	Anthocyanidin synthase (ANS/LDOX)
TT15	AT1G43620	*A. thaliana*	68.6	9.1	5	Biosynthesis	UDP-flavonoid glucosyl transferase (UFGT)
Banyuls (BAN)	AT1G61720	*A. thaliana*	63.5	36.2	22	Biosynthesis	Anthocyanidin reductase (ANR)
TT10	AT5G48100	*A. thaliana*	61.2	55	14	Biosynthesis	Flavonoid oxidase
VvLAR1	GSVIVG01011958001	*Vitis vinifera*	52.9	43.9	8	Biosynthesis	Leucoanthocyanin reductase (LAR)
TT2	AT5G35550	*A. thaliana*	36.8	32.2	8	Regulation	MYB transcription factor
TT8	AT4G09820	*A. thaliana*	56	8.5	17	Regulation	bHLH transcription factor
TTG1	AT5G24520	*A. thaliana*	73	22.3	13	Regulation	WD40 repeat protein
TTG2	AT2G37260	*A. thaliana*	35.4	12.1	12	Regulation	WRKY transcription factor
TT16	AT5G23260	*A. thaliana*	53.6	36.5	17	Regulation	MADS-box transcription factor
TT1	AT1G34790	*A. thaliana*	46.9	22.1	22	Regulation	Zn-finger transcription factor
TT12	AT3G59030	*A. thaliana*	60.6	50.9	16	Transport	MATE vacuolar transport
TT19	AT5G17220	*A. thaliana*	60.7	28	24	Transport	Glutathione-S-transferase
aha10	AT1G17260	*A. thaliana*	82.5	28.9	20	Transport	H+-ATPase proton pump
Bronze2 (BZ2)	GRMZM2G016241	*Zea mays*	79.5	39.8	44	Transport	Glutathione-S-transferase
ZmMRP3	GRMZM2G111903	*Zea mays*	89.4	48.6	11	Transport	MRP anthocyanin transporter
