# Supplementary material for: Dissecting Genome-Wide Association Signals for Loss-of-Function Phenotypes in Sorghum Flavonoid Pigmentation Traits
Source: G3 (Bethesda). 2013 Nov 1;3(11):2085–94. doi: 10.1534/g3.113.008417 (PMC3815067; doi:10.1534/g3.113.008417)
Supplement: Supporting Information [file supp_g3.113.008417_FileS3.pdf]

File S3

SUPPORTING DISCUSSION

Evidence for other tannin loci from small association panel GLM: The secondary peak from the GLM is found at around 66-67 Mb of chromosome 2, with top SNPs at 66.7 Mb (S2_66783467; $p < 10^{-9}$) and 66.0 Mb (S2_66003858; $p < 10^{-9}$). This diffuse association peak colocalizes with several *a priori* candidate genes (Supporting table 2), including putative MYB transcription factor *Sb02g031190*, which is the sorghum ortholog of maize flavonoid regulator *ZmMYB31* (Fornalé *et al.* 2010). With the CMLM the only association peak outside the *Tannin1* region that is significant at the genome-wide threshold is at 6.2 Mb on chromosome 3, which is over one Mb from the nearest *a priori* candidate gene.

To reduce the effects of genetic heterogeneity in the experiment, we controlled for known functional variants in the *Tannin1* gene by (1) including the presence of the non-functional *tan1-a* or *tan1-b* allele as a covariate in the model ("*tan1*-covariate GLM") or (2) carrying out the GWAS using only lines with *Tan1* wildtype alleles in the coding region ("*Tan1*-only GLM"; n = 112). With both approaches, the most significant association peak lie on chromosome 2 between 8.075 Mb and 8.45 Mb. This region is also present in the GLM but falls below the threshold for genome-wide significance. An *a priori* candidate gene *Sb02g006390* (Chr. 2: 8,003,227 - 8,008,714) lies at the edge of, but not directly under, the association peak. *Sb02g006390* is a putative bHLH transcription factor and a co-ortholog of two genes known to control grain tannins, rice *Rc* (Furukawa *et al.* 2007) and Arabidopsis *TRANSPARENT TESTA8* (Nesi *et al.* 2000). This association peak also colocalizes with previously described pigmented testa QTL (Mace and Jordan 2010; Wu *et al.* 2012).

A number of other significant associations are observed using the *Tan1*-only GLM. The second most significant association is at 57.9 Mb on chromosome 3 (S3_57899793; $P = 2x10^{-8}$). The closest *a priori* candidate is a putative leucoanthocyanin reductase (LAR) found 170kb downstream. The next most significant peak spans 1.16 Mb to 1.23 Mb on chromosome 1 (top SNP: S1_1232724; $P = 4x10^{-8}$). Within this peak (and 53kb from the top SNP) is *a priori* candidate gene *Sb01g001230*, a putative Glutathione-S-Transferase and sorghum ortholog of Arabidopsis *TRANSPARENT TESTA19* (Kitamura *et al.* 2004). Finally, even after controlling for known *Tannin1* loss-of-function alleles (*tan1-a* and *tan1-b*) there are still marginally significant association peaks near the *Tannin1* locus (*e.g.* S4_60641625) suggesting that additional loss-of-function alleles for *Tannin1* may exist.

References

Fornalé, S., X. Shi, C. Chai, A. Encina, S. Irar *et al.*, 2010 ZmMYB31 directly represses maize lignin genes and redirects the phenylpropanoid metabolic flux. The Plant Journal 64: 633–644.

Furukawa, T., M. Maekawa, T. Oki, I. Suda, S. Iida *et al.*, 2007 The Rc and Rd genes are involved in proanthocyanidin synthesis in rice pericarp. The Plant Journal 49: 91–102.

Kitamura, S., N. Shikazono, and A. Tanaka, 2004 TRANSPARENT TESTA 19 is involved in the accumulation of both anthocyanins and proanthocyanidins in Arabidopsis. The Plant Journal 37: 104–114.

Nesi, N., I. Debeaujon, C. Jond, G. Pelletier, M. Caboche *et al.*, 2000 The TT8 Gene Encodes a Basic Helix-Loop-Helix Domain Protein Required for Expression of DFR and BAN Genes in Arabidopsis Siliques. The Plant Cell Online 12: 1863–1878.
